# Advancing cancer treatment with nanozyme frameworks: Integrating photothermal, photodynamic, sonodynamic, and chemodynamic therapies

**DOI:** 10.22038/ijbms.2025.80721.17487

**Published:** 2025

**Authors:** Seyed Mohammad Mahdi Rais Sadati, Javad Zamanian, Mohammad Moshiri, Majid Ghayour Mobarhan, Khalil Abnous, Seyed Mohammad Taghdisi, Leila Etemad

**Affiliations:** 1 Pharmaceutical Research Center, Pharmaceutical Technology Institute, Mashhad University of Medical Sciences, Mashhad, Iran; 2 Department of Medicinal Chemistry, School of Pharmacy, Mashhad University of Medical Sciences, Mashhad, Iran; 3 Targeted Drug Delivery Research Center, Pharmaceutical Technology Institute, Mashhad University of Medical Sciences, Mashhad, Iran; 4 Department of Pharmaceutical Biotechnology, School of Pharmacy, Mashhad University of Medical Sciences, Mashhad, Iran; 5 Medical Toxicology Research Center, School of Medicine, Mashhad University of Medical Sciences, Mashhad, Iran; 6 Department of Clinical, Toxicology, Imam Reza Hospital, Mashhad University of Medical Sciences, Mashhad, Iran; 7 International UNESCO Center for Health-Related Basic Sciences and Human Nutrition, Mashhad University of Medical Sciences, Mashhad, Iran; 8 Research Center, Pharmaceutical Technology Institute, Mashhad University of Medical Sciences, Mashhad, Iran

**Keywords:** Cancer, Chemodynamic, Nanozyme, Photodynamic, Photothermal, Sonodynamic, Tumor therapy

## Abstract

Cancer is considered a serious threat to human life and one of the major leading causes of death in the world. As a critical medical challenge in developing and developed countries globally, progress in the design of theranostic nanomedicine is associated with the control of temporal-spatial variability, enhancing the site-specific therapy, and reducing the toxicity to normal tissue. As the primary noninvasive cancer treatment technique, photothermal therapy through radiation absorption in the near-infrared region generates hyperthermia for the ablation of cancerous cells. Photothermal therapy combined with other therapeutic techniques, including chemodynamic, photodynamic, and sonodynamic, has synergistic and enhanced effects on cancer therapy. Nanozymes, as intrinsic multienzyme mimics, can be robust cancer nanotherapeutics owing to the dual effect of catalytic functions and physicochemical advantages of nanomaterials. Nanozymes possess remarkable stability, precise penetrability, exceptional specificity, outstanding recoverability, and minimal toxicity. These attributes make them immensely powerful for therapeutic applications. In light of the significance of multifunctional nanozymes and their increasing focus on catalytic therapy for cancer tumors through reactive oxygen species (ROS), we have compiled a comprehensive overview of recent advancements in various photothermal-based assays utilizing nanozymes. Notably, our analysis reveals that incorporating nanozymes in PTT enhances the generation of ROS, leading to improved therapeutic efficacy against the tumor. In summary, this comprehensive overview highlights the significance of multifunctional nanozymes in advancing photothermal-based assays for cancer treatment. The findings underscore the potential of these innovative approaches to improve treatment precision and effectiveness while reducing adverse effects on healthy tissues.

## Introduction

Cancer is a primary threat to humans, and survival has become a significant challenge in the medical field. Unfortunately, there is a rising trend of cancer-related mortality due to rapid metastasis and low prognosis. Therefore, anticancer therapeutic methods have gained considerable attention all over the world. The routine clinical assays for cancer treatment include surgery, chemotherapy, herbal therapy, radiotherapy, and immunotherapy (1, 2). Nevertheless, these traditional techniques have limitations of surgical trauma, drug resistance, and damage to normal cells. Therefore, they are ineffective in improving patients’ life quality and survival rate (3-6). Besides, the probability of future metastasis and the complete eradication of cancerous tissues through these therapeutic methods are still challenging to achieve (7-10). Hence, developing novel noninvasive treatment strategies benefiting from alleviating side effects is crucial to enhancing the survival rate of patients. To reach this purpose, photothermal cancer therapy and its cooperation with photodynamic, chemodynamic, and sonodynamic assays have been of great interest due to superb controllability and potent tumor-preventative capabilities (11-13). Photothermal therapy is based on materials with high photothermal conversion efficiency that induce cancer cell death through increasing temperature in the cancerous microenvironment. Photodynamic therapy utilizes photosensitizers within cancer cells, which are exposed to light of a specific wavelength. This particular illumination prompts the creation of singlet oxygen and reactive oxygen species (14-16). In sonodynamic therapy, a sonosensitizer agent is activated under ultrasound irradiation, producing reactive oxygen species to destroy tumor cells. Chemodynamic therapy is based on the disproportionation of H_2_O_2_ molecules in cancer cells through Fenton or Fenton-like reactions to produce hydroxyl radicals, harmful reactive oxygen species. In cancer therapy, apoptosis is induced by hydroxyl radicals through the inactivation of proteins, destruction of DNA components, and initiation of peroxidation in phospholipid membranes (17-21). 

In recent decades, nanomaterials with unique biochemical characteristics have been applied extensively for tumor diagnosis and treatment. Nanozymes, as a group of nanomaterials with intrinsic enzyme-like functions, are ideal candidates for tumor therapy due to their remarkable advantages, including simple preparation, high stability, flexible designs, low cost, great endurance toward complicated environments, tunable size, and controllable enzyme-like performance. Besides, they possess enhanced electrical conductivity, high surface-to-volume ratio, superparamagnetic behavior, exceptional fluorescence properties, and spectral shift of optical absorption (22-26). To date, diverse nanomaterials have been employed to obtain efficient photothermal, sonodynamic, chemodynamic, photodynamic therapy, and targeted drug delivery. Nanozymes mainly contain semiconducting polymers (27, 28), carbon nanomaterials (29), metal-organic frameworks (MOFs) (30-32), and metal nanomaterials (33). Some sulfide and disulfide nanomaterials, such as Ag_2_S, Bi_2_S_3_, CdS, MoS_2_, and CuS, have been applied to reach high biocompatibility and sound therapeutic effects (34-37). In addition, stable precious metals and their oxides are superior therapeutic agents due to efficient photothermal conversion and good biocompatibility (38-41). Gold nanoparticles can be used as an anchor for antibodies and pharmacological agents, and also, in sonodynamics, they are an excellent center for cavities, which are safe and have a significant uptake by cells(42). Gold nanoparticles (AuNPs) with superior peroxidase-mimic activity are promising in the cancer therapeutic field through hydroxyl radicals generation at acidic microenvironments, cell apoptosis, and tumor ablation (43, 44).

 Metal oxide nanomaterials with core-shell and yolk-shell structures possess distinctive properties such as interstitial hollow space features and a substantial surface area. These attributes render them exceptionally suitable for precise drug delivery systems, ensuring controlled release while minimizing undesirable consequences (45-48). Carbon-based nanomaterials with the unique physico-chemical advantages of long-term stability, high specific surface area, and favorable conductivity are efficient nanozymes. Mesoporous carbon nanospheres possess large pore volume, uniform size, excellent electrical conductivity, good biocompatibility, and heat generation capability, making them superior candidates for photothermal, sonodynamic, chemodynamic, and photodynamic therapy (49-51). Structural diversity, low cytotoxicity, high specific surface area, biodegradability, facile synthesis on the nanoscale, alternative functionalization via surface chemistry, and good biocompatibility make MOFs good candidates for therapy and drug delivery hosts (52-54). 

**Scheme 1:**
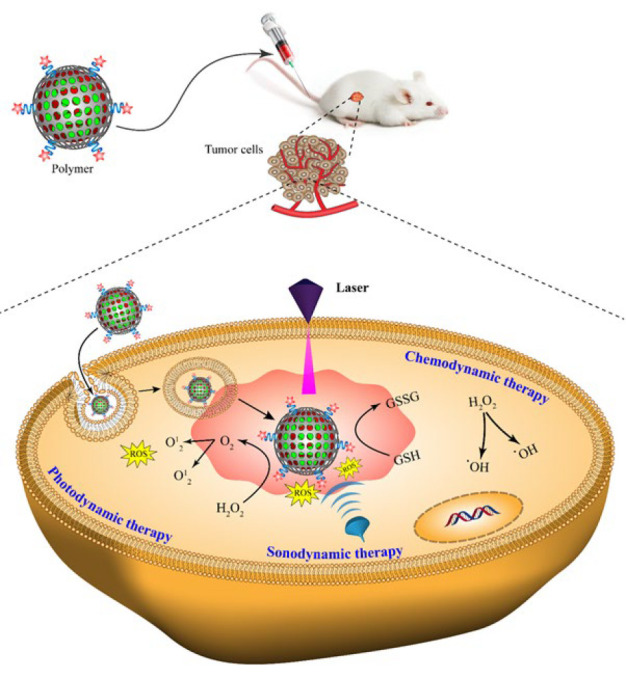
Schematic illustration of the induction of photothermal-combined techniques based on nanozyme frameworks for cancer treatment

Nanozymes have successfully addressed certain limitations commonly associated with natural enzymes, such as their heightened susceptibility to the surrounding environment. The multienzyme activity of nanozymes induces efficient signal changes in the physiological cell microenvironment for catalyzing substrates, producing reactive oxygen species, and ultimately cancerous cell death. This review details the importance and competency of multifunctional nanozymes for reinforcement with photothermal combined therapeutic methods to reach efficient cancer therapy (Scheme 1). 

## Photothermal assays

Photothermal therapy is a cancer treatment method based on converting light energy into heat for localized tumor ablation through photothermal agents with high tissue penetration capability, leading to cancer cell death from apoptosis to necrosis. Photothermal therapy offers the advantages of rapid recovery and excellent control over time (55, 56). When PTA (Photothermal Agent) is irradiated by NIR, two different effects can occur. The light can be scattered or absorbed, which the absorbed light causes electrons to exit the ground state and enter a higher state. In PTA, the electrons return to the ground state by emitting heat following the nonradiation relation path(57).

**Figure 1:**
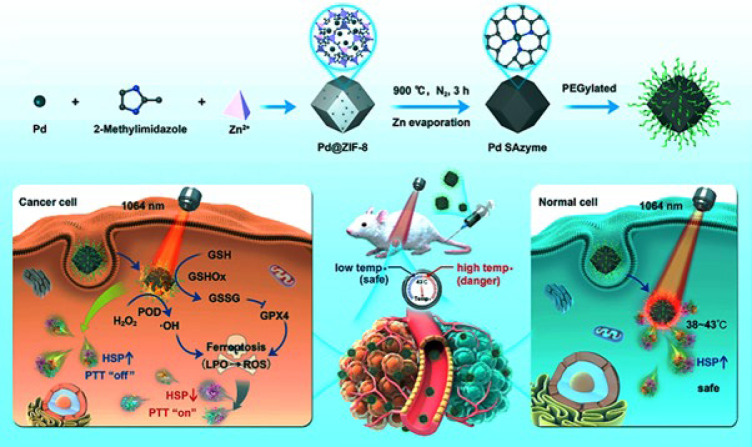
Schematic illustration of the construction and function of Pd SAzyme for cancer therapy

Chang *et al*. (58) fabricated an innovative single-atom nanozyme (SAzyme) with peroxidase- and glutathione oxidase-mimicking roles for ferroptosis-boosted photothermal therapy (Figure 1). The “top-down” stripping of metal nanostructures into single atoms was used to construct Pd SAzymes. In brief, the ultrasmall PdNPs were encapsulated into zeolitic imidazolate framework-8 (ZIF-8), obtaining Pd@ZIF-8 nanocomposite. After three hours of pyrolysis at 900 °C under a nitrogen atmosphere, the Pd single atom nanozyme (Pd SAzyme) was formed for further photothermal therapy. To improve its biocompatibility, the Pd SAzyme surface was modified by PEG molecules. The nanozyme displayed no harmful effects on L929 cells (fibroblast cell line), whereas its ability to eliminate 4T1 cells (breast cancer cell line) increased progressively with the dosage. Meanwhile, in 4T1 cancer cells, peroxidase-mimicking of Pd SAzyme produced reactive oxygen species under NIR-II radiation. Glutathione oxidase-like activity of nanozyme reduced glutathione into glutathione disulfide and further production of glutathione peroxidase4(GPX4) protease, which regulated component of lipid peroxides, and generated the reactive oxygen species for efficient ferroptosis. Subsequently, the Pd SAzyme demonstrated remarkable efficacy in converting light into heat within the near infrared-II region (NIR-II), specifically between 1000 and 1400 nm, during which an infrared camera monitored the temperature. Consequently, it exhibited potent antitumor activity against BALB/c mice bearing 4T1 cancerous xenografts. In normal tissues, Pd SAzyme induced any thermal damage relying on the protective capability of heat shock proteins. The Pd SAzyme could target the 4T1 tumor cells of BALB/c mice by enhancing the permeability and retention (EPR) effect, killing all cancer cells, and eventually elimination through the hepatic excretion pathway. 

Zhu *et al*. (59) introduced a manganese-based SAE (single-atom enzyme) modified PEG with astounding catalytic activity through coordinating manganese to nitrogen atoms in a hollow ZIF-8 skeleton. After etching ZIF-8 nanocubes with tannic acid, a hollow nanostructure was constructed with some tannic acid molecules on its surface. Afterward, H^+^ ions were released from TA and penetrated the ZIF-8 core. Manganese ions were located in the ZIF-8 hollow nanocubes through the ion exchange process. Then, it was pyrolyzed at 900 °C under an argon atmosphere, which was converted into a N-doped carbon skeleton. The Mn/SAE structure was completed by trapping manganese atoms by the N-rich porous carbon. The Mn/SAE possessed outstanding Fenton function with more potency for disrupting the cellular redox balance than conventional MnO_2_ under an 808 nm laser exposure. Mn/SAE catalyzed the H_2_O_2_ conversion to O_2_ molecules based on its catalase activity in 4T1 tumor-bearing BALB/c mice. As a source for generating superoxide radical (·O_2_⁻) reactive species, O_2_ molecules were affected by the oxidase-mimicking function of the nanozyme and produced numerous ·O_2_⁻ radicals. The peroxidase activity of the nanozyme resulted in the generation of hydroxyl radicals through the dissociation of H_2_O_2_ molecules. Effective tumor therapy could be achieved by relying on the photothermal and multi-catalytic activities of the Mn/SAE. Cell apoptosis of Mn/PSAE was about 44.9%, which increased to 68.0% after adding H_2_O_2_. Interestingly, cell apoptosis rose to 85.7% through the combination of irradiation with an 808 nm laser. Combining the nanozyme by laser irradiation decreased the tumor size during the first six treatment days, and the tumor tissues were completely destroyed on the sixth day after treatment.

**Figure 2:**
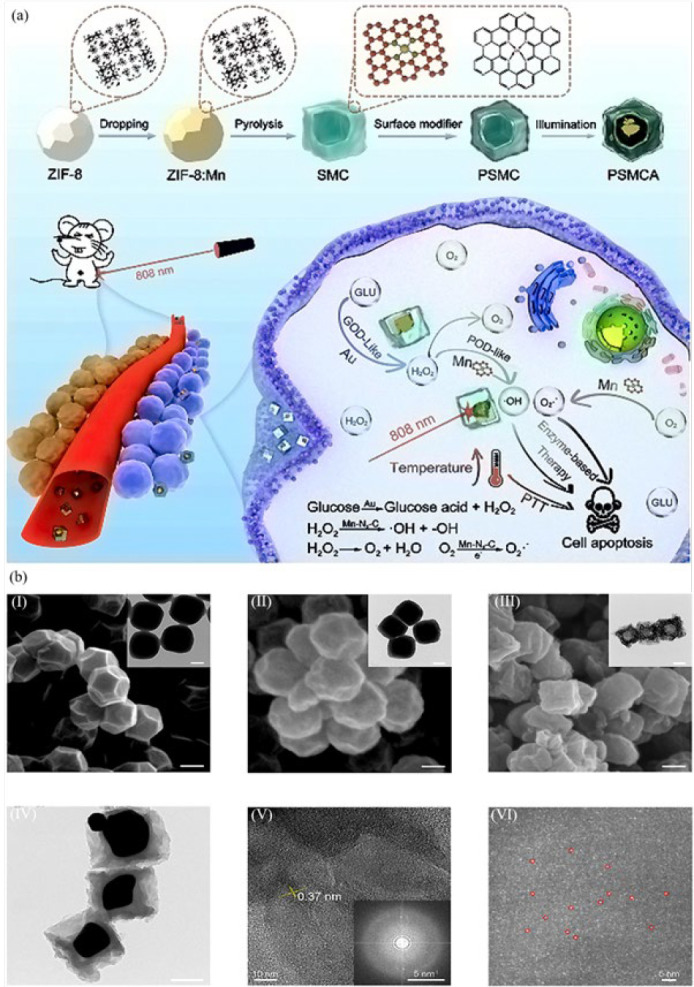
(a) Schematic representation of construction and function of Mn-doped single atom catalyst (SMC) for photothermal cancer therapy. After incorporation and pyrolysis of manganese in the ZIF-8 skeleton, the obtained nanostructure was PEGylated, and gold atoms were embedded into ZIF-8, resulting in the core-shell structure of PSMCA. In the tumors, the photothermal activity of the PSMCA appeared under 808 nm laser irradiation. Based on its gold component, the nanostructure catalyzed glucose to H_2_O_2_ molecules. The peroxidase-like activity of its manganese component produced oxygen-reactive segments for efficient tumor therapy. Transmission electron microscopy (TEM) and scanning electron microscope (SEM) images of the PSMCA components are shown at the bottom; (b) (I) ZIF-8 with delicate dodecahedron structure, (b) (II) manganese involved ZIF-8, (b) (III) manganese-doped SMC with hollow hexahedron, (b) (IV) gold/manganese-doped SMC, (b) (V) high-resolution TEM of manganese-doped SMC, (b) (VI) gold/manganese-doped SMC respectively. Reproduced by permission from Elsevier (60).

Researchers (60) constructed an Mn-doped single-atom catalyst (SMC) modified with PEG and anchoring Au in and on the SMC to obtain (PSMCA) nanozyme as a nano-catalytic tumor therapy with photothermal performance. The fabrication and function of the developed nanozyme are given in Figure 2. First, manganese chloride was incorporated into the ZIF-8 skeleton with a diameter of 250 nm. Following the pyrolysis procedure, manganese ions were fully encapsulated within ZIF-8, resulting in remarkable water solubility due to subsequent PEGylation. After growing gold within the manganese-ZIF-8 skeleton, the complete core-shell structure of the nanozyme was obtained. In HeLa cells (cervical cancer cell line), the PSMCA induced effective photothermal activity under 808 nm laser irradiation. The photo was taken with an Infrared thermal imager to record the temperature. In the presence of the gold component within the nanozyme, cells exhibit oxidase-like activity and produce H_2_O_2_ due to glucose consumption. The manganese component’s peroxidase-like activity facilitates the production of toxic reactive oxygen species, such as hydroxyl radicals, through the overexpression of hydrogen peroxide. This process aims to ensure a proficient therapeutic outcome. The tumor tissues (heart, spleen, liver, kidney, and lung) were damaged and broken significantly under the treatment of the nanozyme. Meanwhile, tumor tissues remain intact after treatment with PBS under laser light irradiation. In addition, after different treatments of tumor tissues for 14 days, treatment with PSMCA under 808 nm laser irradiation showed a significant therapeutic effect by reducing the tumor size compared to the other treatments. 

Jia *et al*. (61) introduced a compact-size AgPd plasmonic blackbody (AgPd PB) nanozyme with boosted photothermal and catalytic effects for cancer therapy. First, palladium chloride acid (H_2_PdCl_4_) was mixed with cetyltrimethylammonium chloride (CTAC). Sodium borohydride (NaBH_4_) as a reducer was added to the mixture to achieve the solution of palladium seeds. In the next step, silver nitrate (AgNO_3_), H_2_PdCl_4_, and ascorbic acid were added to the seed’s solution. After two hours of standing, flower-shaped silver@palladium nanozyme was formed successfully. The nanozyme possessed a wide range of resonance absorption across 400–1300 nm, which proved its great photothermal potency. The IR camera was applied to record the temperature at the tumor site. Based on catalase activity, the nanozyme dissociated H_2_O_2_ into O_2_ molecules to relieve tumor hypoxia. Moreover, it generated significant hydroxyl radicals relying on the peroxidase-catalytic function that minimized heat shock proteins for balancing the photothermal effect. The nanozyme induced high toxicity to HeLa tumor cells. The antitumor function of the nanozyeme was examined in the heart, kidney, liver, lung, and spleen. A negligible amount of the nanozyme remained in the spleen and liver, but it was not detected in other organs. More than 90% of the nanozyme was excreted rapidly through feces (80%) and urine (10%). A 7-fold reduction in tumor size, compared with the control group, indicated the effectiveness of nanozyme for cancer theranostics.

researchers (62) developed an antitumor nanozyme by doping Prussian blue (PB) nanoparticles with rare earth ions, including Yb^3+^, Gd^3+^, and Tm^3+^. Among the synthesized nanozymes, Yb-PB possessed the best photothermal and catalytic function. Doping Yb^3+^ improved the electron transfer property of the nanozyme and, consequently, enhanced its catalytic efficiency. To prevent any harmful side effects caused by the reaction of potential H_2_O_2_ with healthy cells, a protective coating of polydopamine (PDA) targeted towards glutathione was applied to encase the Yb-PB nanozyme. However, PDA could eliminate a part of glutathione for the subsequent generation of reactive oxygen components. The enzymatic function of the nanozyme obtained a significant amount of hydroxyl radical for cancer therapy, fluorescence imaging, and magnetic resonance imaging. The viability of 4T1 cells decreased dose-dependently in the presence of Yb-PB@PDA nanozyme. After treatment by the nanozyme, the survival rate of 4T1 cells decreased significantly, proving the generation of many hydroxyl radicals in the tumor environment. Under 808 nm laser irradiation, the temperature of the mouse tumor site rapidly increased above 56 °C within 2 min, clarifying the good photothermal performance of the nanozyme compared to other treatments. A visual thermography thermal imager was applied to record and monitor the temperature of the aqueous solution.

**Figure 3:**
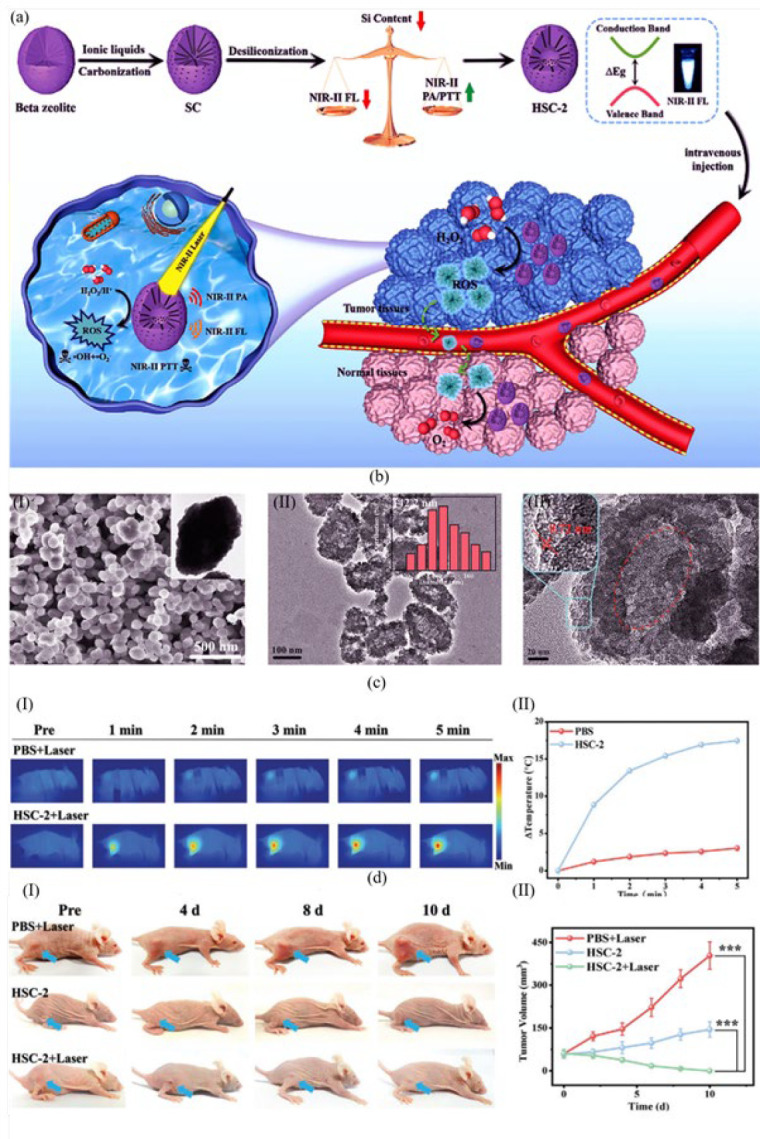
(a) Schematic indication of the HSC-2 nanozyme-mediated photothermal therapy. First, a Beta zeolite skeleton was modified by carbonization and adsorption of ionic liquid. HSC-2 structure was completed after desiliconization. The silicon-carbon ratio in HSC-2 nanozyme determined its photoacoustic and fluorescence emission function. After injection into cancer cells, HSC-2 inhibited the tumor progress by its photothermal function under NIR-II radiation. The peroxidase catalytic activity of the nanozyme obtained a significant number of reactive oxygen species from H_2_O_2_ molecules, effective for tumor therapy. TEM for SC and HSC-2 nanozyme and high-resolution TEM of HSC-2 are indicated at the bottom of the picture (left-to-right). (b) (I) The prepared SC possessed a jujube-like structure (143.7 nm), (b) (II) HSC-2 nanozyme had a uniform morphology with the size of 142.2 nm (b), (III) Hollow structure of the nanozyme has channels with 0.72 nm in size. (c) (I) Thermal photos of tumor-bearing mice (4T1) after treatment with PBS and HSC-2 exposed to laser irradiation (1064 nm) for 5 min. (c) (II) Mean temperatures of tumors treated with PBS and HSC-2. (d) (I) Digital photos of mice from different treatment groups, including PBS+Laser, HSC-2, and HSC-2+Laser, during 10 days. (d) (II) Tumor growth curves of various groups. Reproduced by permission from John Wiley and Sons (63).

Zheng *et al*. (63) designed an HSC-2 (NIR-II PA/NIR-II FL imaging-adjustable nanozyme) to achieve accurate photothermal and catalytic tumor treatment using a zeolite-carbon structure. As depicted in Figure 3a, the initial structure of HSC-2 was formed by carbonization and ionic liquid 1-butyl-3-methylimidazolium bromide (BMIMBr) adsorption on a Beta zeolite skeleton to achieve SC. The carbonization process induced a superb NIR-II emission performance to the nanostructure. After desiliconization of the nanostructure, the complete skeleton of the HSC-2 nanozyme was formed. By manipulating the silicon-carbon ratio within its structure, one can effectively adjust the dual functionality of HSC-2 nanozyme, specifically regarding NIR-II photoacoustic and NIR-II fluorescence emission. In 4T1 tumor-bearing BALB/c mice, NIR-II photothermal function of HSC-2 inhibited tumor growth. HSC-2 nanozyme utilizes its peroxidase activity to convert H_2_O_2_ molecules into reactive oxygen species under 1064 nm laser irradiation, resulting in effective tumor therapy. According to Figure 3cII, there is a significant variation in the temperature of the tumor area. The HSC-2+Laser group experiences an approximate change of 17.46 °C, while the PBS group shows a difference of around 2.2 °C. This indicates their respective effectiveness in inducing photothermal killing. As depicted in Figure 3dI, in the tumor HSC-2+Laser group, the tumor was burnt and left a black scar. Strikingly, compared to the HSC-2 and PBS groups, in the HSC-2+Laser group, the tumor completely disappeared after 10 days (Figure 3dII). 

Tang *et al*. (64). introduced a novel ceria-loaded gold@platinum nanozyme for dual catalytic-photothermal tumor therapy. First, mesoporous gold@platinum nanosphere was synthesized by reducing gold (III) chloride trihydrate and potassium tetrachloroplatinate (II) by ascorbic acid under an ultrasonic reaction. Ceria (CeO_2_) nanoparticles were obtained through an inverse micellar process. The CeO_2_/Au@Pt synthesis was completed by sonicating and stirring the gold@platinum nanosphere with ceria. Subsequently, noticeable dispersibility was obtained by modifying CeO_2_/Au@Pt with SH-PEG. The average size of dendritic gold@platinum nanosphere and ceria nanoparticles was 50 ± 10 nm and 3 ± 1 nm, respectively. With the inclusion of ceria in the nanosphere, the size increased to 80 ± 20 nm. In the HeLa cancer cells, the nanozyme induced high photothermal effects under 808 nm laser radiation based on the exceptional absorption of gold@platinum nanosphere in the NIR bio-window. Changes in the temperature were monitored by Infrared thermography. Besides, the nanozyme-induced peroxidase-like activity relied on the catalytic properties of ceria nanoparticles and generated many hydroxyl radicals for tumor therapy. 

In their study, Wang and colleagues (65) presented a hydrogel that encapsulates camptothecin (CPT) and pyrite (FeS_2_), serving as an exceptional nanozyme for chemotherapeutic drugs. This innovative approach combines photothermal therapy with nanozyme-catalyzed treatment involving peroxidase (POD) and glutathione oxidase (GSH-OXD) activities. To form an injectable composite hydrogel (CFH) system, agarose hydrogel was mixed with chemotherapeutic drug (CPT) and FeS_2_ nanozyme and stirred at 60 degrees. The hydrogel content of the nanozyme could regulate the release of CPT and FeS_2_. After 808 nm NIR laser irradiation, FeS_2_, as the photothermal agent, converted light energy into heat. After the release to the CT26 tumor cell (colorectal carcinoma cell line) environment, CPT produced many H_2_O_2_ molecules through its nicotinamide adenine dinucleotide phosphate oxidase (NOX) activity. Then, FeS_2_ catalyzed H_2_O_2_ molecules to hydroxyl radicals and depleted glutathione into glutathione disulfide that amplified oxidative stress. With the high durability of nanozyme in cancerous cells, the release of drugs and their antitumor functions could be adjusted. The nanozyme-induced glutathione oxidase and peroxidase activities are efficient for catalytic cancer treatment. The developed nanozyme showed strong antitumor properties without damaging healthy tissues. 

Wu *et al*. (66) decorated Bi_2_Te_3_ nanosheets with Au/Pd bimetallic nanoparticles homogeneously to obtain Bi_2_Te_3_-Au/Pd nanozyme as a second near-infrared (NIR-II) photothermal-nanocatalyst. To prepare Bi_2_Te_3_-Au/Pd nanocatalysts (BAP NCs), Au NPs and Pd NPs were in order deposited on the Bi_2_Te_3_ nanosheets and consequently modified with PEG. The nanosheets provided a high surface area for loading many gold and palladium, serving as the active sites. In 4T1 tumor-bearing mice, a photothermal conversion efficiency of 59.0% was achieved by the nanozyme under 1064 nm laser radiation. A thermal imaging camera was then used to record the changes in the temperature. The nanozyme exhibits a 2.24-fold increase in the generation of oxygen molecules when operating at 45 °C compared to room temperature, owing to its remarkable ability to enhance catalytic reaction rates through significant thermal effects. Due to the enhanced catalytic performance of the Bi_2_Te_3_-Au/Pd nanozyme, which exhibited a 1.38-fold increase in activity compared to Bi_2_Te_3_-Au alone, it can be concluded that the alloy structure played a crucial role in this improvement. Based on its peroxidase- and catalase-like function and glutathione depletion, the nanozyme afforded efficient hypoxia suppression and ferroptosis. Also, the *in vitro* and *in vivo* experiments proved the Bi_2_Te_3_-Au/Pd nanozyme’s excellent capability for suppressing tumor growth via immunogenic cell death.

Zhao *et al*.(67) fabricated gold nanostars (GNS) with dual catalytic and photothermal capability to reach the synergic effect. Gold nanostars were prepared using a safe, facile, and surfactant-free one-step approach using poly (γ-glutamic acid) (γ-PGA) as a biodegradable medium. Their star-like morphology was due to the binding of the carboxyl group of γ-PGA to gold by coordination and ionic interactions(68-70). The synthesis method resulted in a precise size of 191 nm. Due to the comparable POD-like activity of Fe_3_O_4_ nanozym to GNS, it was used as a control group to show the combined cascade catalyze therapy. The result of an *in vitro* study on the 4T1 cell line showed that GNS groups in combination therapy had better performance in treatment than Fe_3_O_4_, GNS. Among all GNS groups (GNS+H_2_O_2_, GNS+NIR and GNS+NIR+H_2_O_2_) in a dose-dependent manner, GNS+NIR+H_2_O_2_ showed better tumor therapy effect. In 4T1 tumor-bearing mice, the high temperature of 75 °C was generated in 10 min under NIR irradiation. A thermocouple thermometer and thermal camera were then applied to record the temperature. A photothermal conversion efficiency of 35.7% was calculated for the gold nanostars, which was higher than that for spherical gold. The nanozyme simultaneously possessed glucose oxidase- and peroxidase-like activities, which catalyzed glucose into H_2_O_2_ molecules and then decomposed them to toxic hydroxyl radicals. The catalytic activity of gold nanostar cloud can be amplified 1.4 fold in combination with its photothermal effect. Gold nanostar nanozyme shows excellent biocompatibility, remarkable photoacoustic and infrared thermography capabilities, and an impressive antitumor rate of 97.0%, making it a highly promising candidate for anticancer therapy.

Researchers (71) designed a multifunctional nanozyme with glutathione oxidase-, catalase-, and peroxidase-mimicking properties for catalytic treatment of tumor calls. Cobalt (Co^2+^) and lanthanum (La^3+^) ions were doped into Prussian blue (PB) as a photothermal agent with potent charge transfer absorption in the NIR region. Co/La-PB was covered by MOF-199 as a copper (Cu^2+^)-based scaffold to load glutathione oxidase molecules. After collapsing MOF-199 from the surface of PB in the acidic environmental conditions of cancerous cells, Cu^2+^ ions were released for the continuous consumption of glutathione. Besides, the presence of metal ions with a high oxidation state accelerated the glutathione oxidase function of the nanozyme. Glutathione oxidase molecules were detached from the MOF-199 surface, which could interact with glucose to produce H_2_O_2_ and gluconic acid. The consumed O_2_ molecules in the reaction with glucose were resupplied by the catalase activity of Co/La-PB@MOF-199. The peroxidase-like function of the nanozyme induced many hydroxyl radicals by catalyzing H_2_O_2_. The survival rates of the normal and cancerous cells (293T and 4T1) treated with the nanozyme under NIR irradiation were 85% and 55%, respectively. The nanozyme showed negligible toxicity against normal cells. *In vivo* studies also showed excellent tumor suppression in mice treated with Co/LaPB@MOF-199/GOx under NIR irradiation compared with Co/LaPB@MOF-199/GOx. However, due to POD-like (Peroxidase like), CAT-like (catalase like), and GPx-like (glutathione oxidase like) activity of nanozyme, tumor growth of Co/La-PB was slower than in mice treated with PBS. 

**Figure 4:**
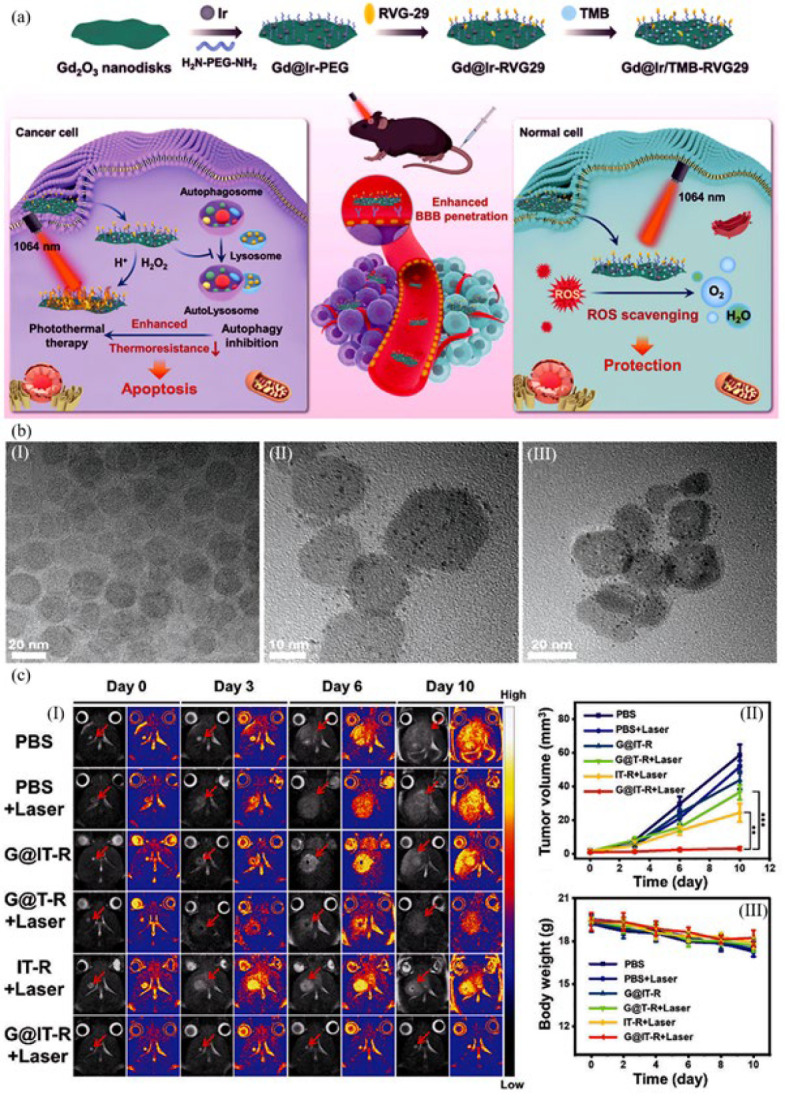
(a) Schematic representation of the formation and function of Gd_2_O_3_@Ir/TMB-RVG29 for the photothermal therapy of cancer cells. Gd_2_O_3 _nanodisks were synthesized based on the decomposition of gadolinium-acetate precursors and modified by H_2_N-PEG-NH_2_ and PVP. Ir nanodots, the photothermal segment, were immobilized on the surface of nanodisks. After attaching the RVG29 peptide to the modified nanodisks, the prepared nanozyme could penetrate through BBB and target gliomas. In the cancer cells with high H_2_O_2_ concentration, the peroxidase-like activity of the nanozyme amplified the TMB chromogenic reaction for photothermal therapy. (b) TEM for Gd_2_O_3_ nanodisks, Gd_2_O_3_@Ir-PEG and Gd_2_O_3_@Ir/TMB-RVG29 is exhibited at the bottom of the picture (left-to-right). The TEM image of the Gd_2_O_3_ nanodisk proved its uniform morphology with a dimension of about 20 nm. Gd_2_O_3_@Ir-PEG possessed the grown Ir nanodots on the uniform Gd_2_O_3_ substrate. Also, Gd_2_O_3_@Ir/TMB-RVG29 had a diameter range of 20–30 nm. (C)(I) Representative T2-weighted MRI photos of mice of the brains of mice with orthotopic gliomas in each group (red arrow indicates tumor area). (C)(II) Tumor volume in different groups. (C)(III) Change curve in average body weight of various groups. Reproduced under the terms of the Creative Commons CC BY license, published by John Wiley and Sons (72).

Yin *et al*. (72) designed a robust nanozyme based on Gd_2_O_3_@Ir/TMB-RVG29 hybrids for targeting glioma primary cancer cells with two purposes, including eliminating inflammation and tumor-specific PTT. Figure 4 illustrates the synthesis, function processes, and hydrodynamic characterization of the nanozyme. Ultra-narrow Gd_2_O_3_ disks with a relatively uniform dimension of about 20 nm were synthesized by dissociating gadolinium-acetate precursors. Then, H_2_N-PEG-NH_2_ and polyvinyl pyrrolidone (PVP) were attached to the nanodisks’ surface, improving their biocompatibility and stability. Ir nanodots were fixed on the surface of modified nanodisks as the activating photothermal agent. The nanodisks were then modified by RVG29 peptide through the reaction with the crosslinkers, which prepared them for penetrating through the blood-brain barrier (BBB) and targeting gliomas. 3,3′,5,5′-tetramethylbenzidine (TMB) molecules as the photothermal agent were loaded on the nanozyme surface by interacting with the PVP hydrophobic domain. With the specific binding of RVG29 peptides to the nicotinic acetylcholine receptors placed on the external surface of glioma cells, the nanozymes could be internalized into the cells by passing BBB. In the GL261 tumor cells (glioma cell line), the nanozyme, through its peroxidase-like function, triggered the amplification of the TMB chromogenic reaction for photothermal therapy under 1064 nm laser irradiation. The IR thermal camera recorded and monitored the temperature changes. In the healthy cells (heart, kidney, spleen, lung, and liver), Gd_2_O_3_@Ir/TMB-RVG29 nanozyme indicated good biosafety by preserving their viability over 80%. According to the findings presented in Figure 4c, neither laser nor nanomachines alone demonstrated any therapeutic efficacy during the *in vivo* study. In the G@T-R+Laser group, the lack of Ir nanozyme prevented the activation of the photothermal prodrug, leading to an insignificant antitumor effect. Surprisingly, the G@IT-R+Laser group demonstrated excellent suppression in the growth of orthotopic glioma. The body weight of mice in all groups remained almost unchanged, indicating the biosafety of the nanomaterials. 

## Photothermal-sonodynamic assays

Sonodynamic therapy is a cancer treatment method that combines sonosensitizers with low-intensity ultrasound. With the advantages of deep tissue penetration, noninvasiveness, good patient compliance, low cost, high accuracy, and few detrimental reactions, sonodynamic therapy is suggested to be more effective than photodynamic assays (73, 74). The SDT mechanism still needs to be fully understood. The ultrasound wave works through a mechanism called cavitation. Cavitation works through the interaction between ultrasound waves and the aqueous surrounding liquid, making cavities near the cell membrane and increasing drug penetration in the cell. This cavitation makes microtubules, bringing the sonosensitizer into an excited state, generating ROS, which causes cell toxicity (75).

**Figure 5:**
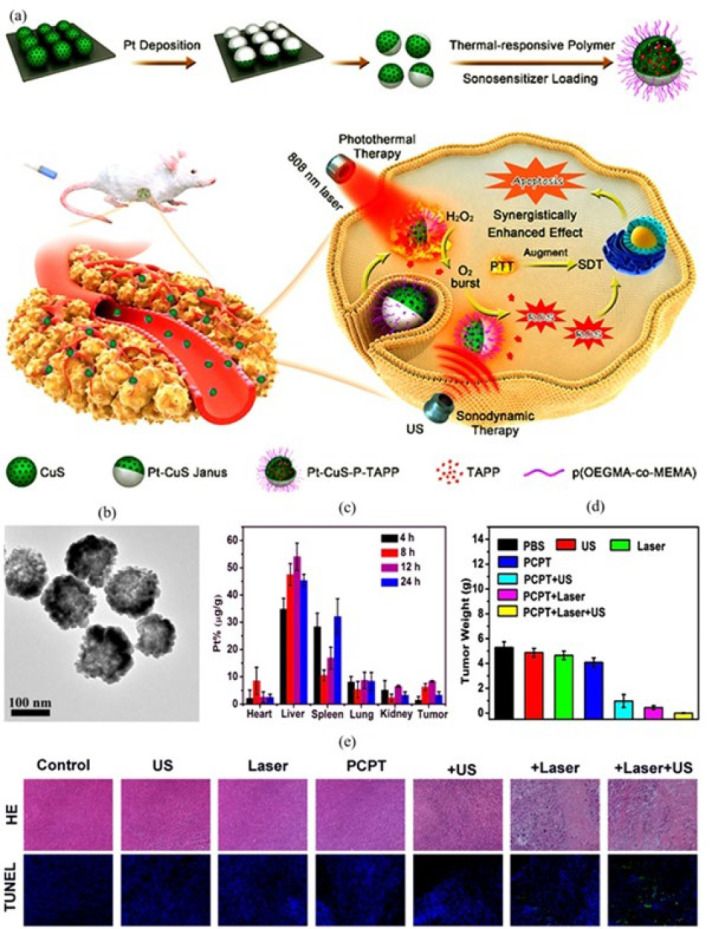
(a) Schematic representation of the synthesis and antitumor action of Pt-CuS Janus nanoenzyme. CuS nanospheres were fixed on a silicon wafer. Pt element covered the top part of CuS NPs. The constructed Pt-CuS Janus were released in water by sonicating in water. The temperature-sensitive copolymers were immobilized on the Janus surface. TAPP sonosensitizer molecules filled the hollow CuS NPs. After intravenous injection of the nanoenzyme into CT26 tumor-bearing mice, the generated heat under 808 nm laser irradiation resulted in the release of TAPP for sonodynamic therapy. The catalytic activity of Pt-CuS enhanced the O_2_ level for further generation of reactive oxygen species for tumor-cell apoptosis. (b) TEM of Pt-CuS Janus, showing its heteronanostructure. (c) Biodistribution of Pt in tumor and main organs in different time intervals after intravenous injection of PCPT. (d) Tumor weights of mice after various treatments. (e) H&E and TUNEL staining in the tumor site after different treatments. Reproduced by permission from the American Chemical Society (76).

Liang *et al*. (76) presented a noninvasive smart architecture of hollow Pt-CuS Janus for efficient cancer therapy utilizing the synergic effect of photothermal and sonodynamic assays (Figure 5a). The synthesized hollow CuS nanoparticles (CuS NPs) were spread spherically on a piece of silicon wafer that formed a monolayer. The upper surface of CuS NPs was covered by a layer of catalytic platinum (Pt) element through a sputter deposition approach. The hydrodynamic characterization of Pt-CuS can be seen in Figure 5b. With sonicating in deionized water, the synthesized Pt-CuS Janus was released in water. Thiol-terminated p(OEOMAco-MEMA) copolymers as the temperature-sensitive molecules were immobilized on the Pt-CuS Janus through Cu-S and Pt-S bonds. Hence, the nanoenzyme activity and drug release percentage could be intelligently controlled by temperature. The theranostic nano-Janus structure was completed by loading tetra-(4-aminophenyl) porphyrin (TAPP) molecules as a sonosensitizer agent, with excellent biosecurity and benefit of positive charge inside the hollow region of CuS NPs to form PCPT. The hollow CuS NPs provided a massive cavity for loading many TAPP as a sonosensitizer. The Pt element, possessing the ability to enhance the local electric field, successfully enhances photothermal activity with exceptional efficiency. Moreover, its superior nanozyme activity induced the high-throughput catalytic dissociation of the innermost overexpressed H_2_O_2_ to O_2_ molecule. By the effective role of the temperature-sensitive copolymer, the generated heat under 808 nm laser irradiation induced the release of TAPP. It accelerated the catalytic activity of Pt-CuS Janus to boost the O_2_ level for further sonodynamic therapy. As a result, efficient cancer therapy could be achieved by overcoming tumor hypoxia and producing cytotoxic reactive oxygen species in CT26 cancer cells. With the synergic effect of photothermal and sonodynamic therapy, the Janus nanoenzyme could eradicate the CT26 tumor cells with no reoccurrence. Most of the NPs were detected in the spleen and liver, proving the inevitable uptake of the nanozyme by the mononuclear phagocyte system (Figure 5c). The superb therapeutic safety guarantees the efficiency of the synthesized nanoenzyme for clinical applications. The decrease in tumor weight proved the therapeutic effect of PCPT NPs, especially in combination therapy with laser and ultrasound (US) (Figure 5d). The HE (hematoxylin and eosin) and TUNEL staining demonstrated obvious changes in cell status and remarkable damages only in PCPT with the US under the 808 nm laser irradiation group. 

A study (77) introduced a novel antitumor strategy by using a multifunctional hydrogel containing PB nanozyme and chlorin e6 (Ce6) sonosensitizer for simultaneous photothermal and sonodynamic therapy. The nanozyme was prepared by a simple hydrothermal method by encapsulating nanozyme PB and the sonosensitizer chlorin e6 (Ce6) into a low-melting agarose hydrogel to form PB+Ce6@Hy. Following the administration of the hydrogel through a local injection into the 4T1 tumor cells, an 808 nm laser was utilized to deliver light energy. This light energy was absorbed by the PB component and subsequently transformed into heat energy, which prompted the liberation of both PB and Ce6 molecules. By irradiation the nanozyme for 10 min under 808 nm laser (0.5 W cm^-2^), the tumor temperature surged to nearly 48 °C. PB proceeded with the catalysis of endogenous H_2_O_2_ conversion to O_2_ molecules and promotion of Ce6-mediated sonodynamic therapy. The hydrogel that has been developed can generate a significant quantity of reactive oxygen species. This characteristic renders it highly effective in suppressing the growth and spread of tumors by eliminating cancerous cells, all while avoiding harmful effects on the body’s normal physiological processes. After the treatment by the nanozyme, the mice organs (heart, kidney, spleen, lung, and liver) collected and analyzed showed no short-term toxicity or side effects from the treatment method. The size stability of nanozyme for a week proved its high stability for long-time therapy. With excellent biocompatibility, biodegradability, and high sensitivity to light stimuli, the nanozyme possessed great potential for cancer therapy.

## Photothermal-chemodynamic assays

Chemodynamic therapy is one of the most effective approaches to treating cancer based on the conversion of internal hydrogen peroxide to lethal hydroxyl radicals through the Fenton or Fenton-like reactions that lead to the destruction of the cancer cells (78, 79). Oxidation of ferrous iron (Fe^2+^) to the frick iron (Fe^3+^) in the presence of hydrogen peroxide as an oxidation agent. Reactions that start with other low valent transition metal complexes like iron are called Fenton-like reactions such as cu^+^(80). Combination of photothermal assays, and chemodynamic methods can induce an effective way to treat the cancer (81).

**Figure 6:**
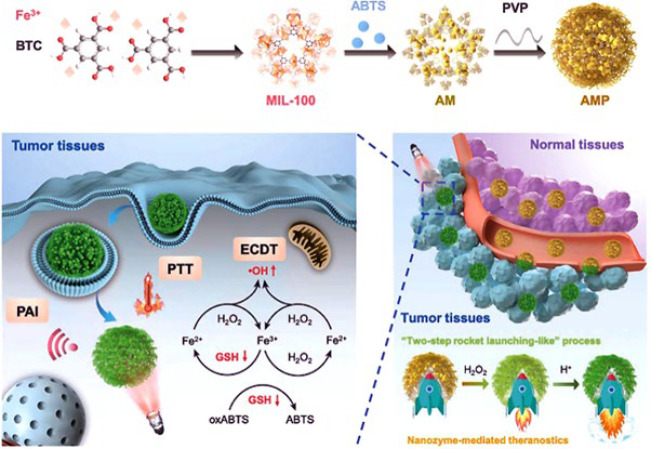
Schematic indication of the nanozyme-mediated photothermal and chemodynamic therapy by the MIL-100 skeleton-based nanoreactor

Liu *et al*. (82) successfully developed a nanoreactor utilizing the MIL-100 MOF as an exceptional nanozyme with peroxidase-like characteristics. This innovative creation holds immense potential for simultaneously conducting photothermal and chemodynamic therapies, specifically targeting tumor theranostics. Figure 6 indicates the components and function of the constructed nanoreactor. At first, 2,2′-azino-bis (3-ethylbenzothiazoline-6-sulfonic acid) (ABTS) was loaded into MIL-100 skeleton, a coating of PVP layer completed the nanoreactor structure to form the final structure of AMP (ABTS@MIL-100/PVP) nanoreactors (NRs). The constructed nanozyme followed three significant properties: first, MOF MIL-100 can act as a peroxidase-like nanozyme and as a carrier for ABTS. Second, AMP NRs can follow two effective performances, including photothermal therapy (PTT) property and photoacoustic imaging (PAI) due to activating by H_2_O_2 _and amplifying in an acidic tumor microenvironment. Finally, AMP NRs can perform chemodynamic therapy through the Fenton reaction and down-regulation of intracellular GSH levels. The nanozyme characteristic of the nanoreactor for photothermal therapy was activated in the presence of H_2_O_2_ and amplified in the acidic microenvironmental conditions of the 4T1 tumor cells. The peroxidase-like activity of MIL-100 resulted in the generation of hydroxyl radicals and disruption of GSH, necessary for efficient enhanced chemodynamic therapy. Besides, MIL-100 could catalyze the oxidation of ABTS in the presence of H_2_O_2_, making the nanoreactor relevant for photoacoustic imaging. The nanozyme-mediated reactor exhibits specificity towards tumor cells without causing harm to normal tissues, thanks to its reliance on H_2_O_2_ and acidic conditions. Based on MTT assay results, only the AMP/H_2_O_2_/NIR group showed significant cytotoxicity against 4T1 cells (at the concentration of 200 µg ml^-1^ that leads to killing about 73% of cancerous cells). No apparent damage or inflammatory lesions in the heart, kidney, spleen, lung, and liver after treatment confirmed the low toxicity of the AMP NRs. In addition, negligible changes in the body weight of mice confirmed the high biocompatibility of the designed nanozyme. 

Qi *et al*. (84) introduced smart plasmonic nanozymes based on carbon dots using Ag/Au bimetallic nanoshells for simultaneous chemodynamic and photothermal therapy of tumor cells. The nanozyme exhibits enhanced catalytic performance due to the presence of carbon dots, which demonstrate a quantum size effect and contribute to an increase in active sites on the surface of the nanozyme. The multifunctional nanozyme included carbon dot-designed Ag/Au bimetallic nanoshells (abbreviated CAANSs). Carbon dots at the size of 0.04 mg ml^-1^ were adsorbed on the Ag/Au nanoshells and acted as efficient photothermal catalysis. CAANSs with more biocompatibility than the Ag/Au nanoshells provided superior photostability and photothermal properties. Following the administration of the CAANSs nanozyme through local injection into HeLa tumor cells and subsequent exposure to an 808 nm laser, a catalytic reaction occurs within the cellular environment. This reaction effectively converts intracellular H_2_O_2_ into reactive oxygen species, primarily focusing on superoxide anions. The acidic nature of this environment further enhances these reactions and ultimately destroys cancer cell DNA. Due to the overproduction of tryptophan (Trp) in plasmonic photothermal therapy, it could participate in an oxidative stress process during cell apoptosis. Trp was decomposed to H_2_O_2_ molecules, which ultimately formed superoxide anions and the destruction of cancer cells under the catalytic process. With an apoptosis of 95%, CAANSs induced an effective catalytic function for cancer treatment.

Researchers (85) developed a simple hybrid of natural tannic acid polyphenol (TA) and ammonium metavanadate (NH_4_VO_3_) with the synergistic effect of photothermal-chemodynamic therapy. TA, an essential micronutrient, can be found in medicinal herbs and vegetarian foods. It exhibits exceptional affinity to bind with metal ions of significant valence. Hence, the utilization of TA-VOx nanosheet enables a notable improvement in absorbing near-infrared radiation, which can then be efficiently converted into thermal energy. 808 nm laser (1.5 W cm^-2^) was applied to examine the photothermal properties of the nanozyme. In comparison with the free TA and NH_4_VO_3_, the TA-VOx nanosheet possessed evident absorption in the broadband NIR region from 600 to 1000 nm, making it a potent phototherapeutic nanoagent. The presence of valence vanadium oxide in the constructed nanosheet provided its superior peroxidase-like activity, which achieved reactive oxygen species for tumor therapy. The catalytic activity of the constructed nanosheet was proved by altering the TMB to a bluish oxidized form in the presence of hydroxyl radical. Indeed, the capability of the TA-VOx nanozyme for the generation of reactive oxygen species was examined in the mouse and human breast cancer cells (4T1 and MDA-MB-231, respectively) by using 2,7-dichlorofluorescein diacetate (DCFH-DA) with high green fluorescence in the oxidized form. The cells emitted green fluorescence after treatment by the nanozyme, while they induced no fluorescence emission in the presence of only H_2_O_2_. The *in vivo* therapeutic capability of the nanozyme was successfully proved in the 4T1-tumor-bearing mice animal model. The great enzyme catalytic activity and NIR absorption of the TA-VOx nanozyme make it a promising agent for photothermal and chemodynamic cancer therapy. 

**Figure 7:**
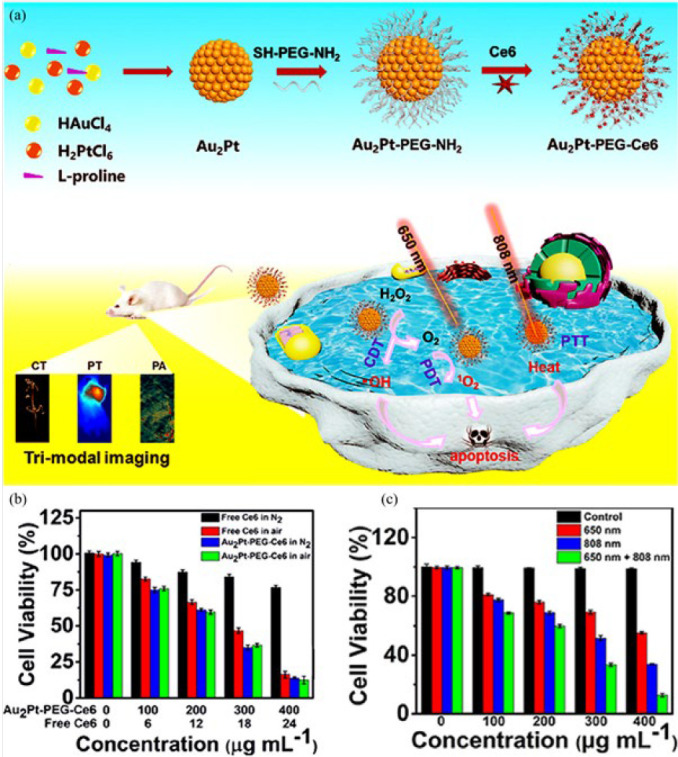
(a) Schematic representation of Au_2_Pt-PEG-Ce6 nanozyme for multiple therapeutic aims. After mixing AuCl_4_^-^ and PtCl6^2-^ with ascorbic acid, Au_2_Pt spheres were generated to immobilize SH-PEG-NH_2_. Ce6 was conjugated to Au_2_Pt-PEG-NH_2_ via an amidation reaction, creating a multifunctional Au_2_Pt-PEG-Ce6 nanozyme for cancer therapy. With high adsorption under 808 nm laser irradiation, the nanozyme was efficient for photothermal therapy. Utilizing its catalase-like activity, the nanozyme transformed O_2_ molecules into singlet oxygen (¹O₂) for photodynamic therapy. Besides, numerous hydroxyl radicals were obtained by the peroxidase-like function of Au_2_Pt-PEG-Ce6 for efficient chemodynamic therapy. (b) Cell viability of different free Ce6 and Au_2_Pt-PEG-Ce6 concentrations against Hela cells to compare their PDT effects under nitrogen and air. (c) The cell viability of Hela cells treated with Au_2_Pt-PEG-Ce6 after 5 min with 808 nm, 650 nm, or both 808 and 650 nm laser irradiation at different concentrations (0, 100, 200, 300, and 400 μg ml^-1^). Reproduced by permission from Elsevier (86).

Wang *et al*. (86) presented Au_2_Pt-PEG-Ce6 nanoformulation with multiple therapeutic functions using synergistic phototherapy and chemodynamic therapy (Figure 7a). First, AuCl_4_^-^ and PtCl_6_^2-^ were reduced in the presence of ascorbic acid to produce Au_2_Pt spheres. To attach Ce6 molecules as a photosensitive agent, SH-PEG-NH_2_ was immobilized on the surface of the spheres through the interaction(87, 88) of the sulfhydryl segment with Pt and Au atoms. Then, Ce6 was attached to Au_2_Pt-PEG-NH_2_ via the carboxyl group in the amidation reaction. With potent absorption in the NIR region, the synthesized nanozyme could be efficient for photothermal therapy and photoacoustic imaging. After an injection to the HeLa tumor cells, the nanozyme with catalase-like activity converted O_2_ molecules into singlet oxygen (^1^O_2_) under 650 nm laser irradiation, resulting in impressive cancer photodynamic therapy. Furthermore, the peroxidase-like characteristics of this substance contributed to an amplified production of hydroxyl radicals, which were utilized in chemodynamic therapy. The PDT effect of Au2Pt-PEG-Ce6 against HeLa cells was due to its significant O_2_ generation capacity through over-expression of H_2_O_2_ (Figure 7b). Upon exposure to both laser irradiations (650 and 808 nm), the cell viability of Au2Pt-PEG-Ce6 (400 μg ml^−1^) treated HeLa cells decreased to 12%, which was much lower than exposure to each of them (Figure 7c). Based on superiorities of easy fabrication, biocompatibility, excellent stability, and cost-effectiveness, the Au_2_Pt-PEG-Ce6 nanozyme showed versatile application prospects, such as photoacoustic imaging and multiple photothermal/photodynamic/chemodynamic therapy.

**Figure 8:**
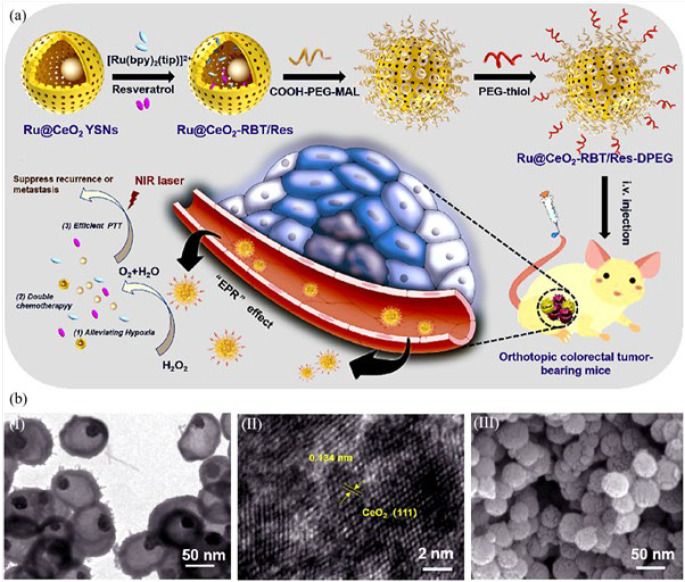
(a) Schematic illustration of fabrication and function of Ru@CeO_2_-RBT/Res-DPEG nanozyme for photothermal and chemodynamic therapy of colorectal tumor cells

Zhu *et al*. (89) fabricated Ru@CeO_2_ yolk-shell nanozymes (Ru@CeO_2_ YSNs) with superb light-to-heat exchange efficiency and catalytic function for antitumor therapeutic aims. Figure 8 depicts Ru@Ce_3_O_4_/CeO_2_ YSNs obtained through a redox reaction of a mixture containing Co^2+^ cation, NH_3_.H_2_O, RuCl_3_, and Ce(NO_3_)_3_. Ru@CeO_2_ YSNs were produced by adding Ce^3+^, followed by subsequent annealing and crystallization. Resveratrol (Res) and ruthenium (RBT) antitumor drugs were dual-loaded in the YSNs using a double layer of polyethylene glycol to construct a biocompatible Ru@CeO_2_-RBT/Res-DPEG drug delivery system. After establishing CT26, a tumor model in BALB/c mice, the nanozyme was used to catalyze H_2_O_2_ to oxygen molecules for further photothermal therapy and chemotherapy under 808 nm NIR laser irradiation. Besides, Ru@CeO_2_-RBT/Res-DPEG induced the antitumor effects through the release of Res and RBT drugs. Excitingly, in comparison with the other groups (saline, RBT/Res+NIR, Ru@CeO_2_ YSNs+NIR, and Ru@CeO_2_-RBT/Res+NIR NIR), only Ru@CeO_2_-RBT/Res-DPEG+ NIR group demonstrated inhibition effect in metastasis of tumor cells. *Notably, the* metastatic regions were smaller than 1-2 mm, which were difficult to detect by routine biopsy. The constructed YSNs are efficient photothermal and chemodynamic therapeutic components that inhibit metastasis formation and recurrence of colorectal tumor cells. 

Jana *et al*. (90) introduced a trimetallic alloy nanozyme with peroxidase activity in circumneutral pH conditions for efficient chemotherapy and photothermal therapy. The alloy nanozyme included Cu, Pd, and Fe metals (PCF) and an outer layer of polyethylene glycol and dopamine, which decorated poly-(isobutylene-alt-maleic anhydride) (DOPA-PIMA-PEG). The presence of transition metals provided the highly efficient production of cytotoxic hydroxyl radicals through the Fenton reaction. In the 4T1 cancer cells, the glutathione peroxidase-like function of the injected nanozyme caused a reduction in the self-antioxidant defense due to depletion of the over-generated glutathione. Under 808 nm NIR irradiation, the photothermal effect of the metallic nanozyme accelerated the generation of hydroxyl radicals for chemodynamic therapy. During the application of ultrasonic radiation, the enzymatic system initiated the Fenton reaction and subsequent production of reactive oxygen species. This process facilitated mass transfer at active catalytic sites within the designed nanozyme, ultimately leading to chemodynamic therapy. Through MTT assay on the 4T1 cell line, in the hypoxia stimulated condition under US and 808 nm laser irradiation, the cell killing efficiency of PCF-a NEs reached 94.4% and 94.2%. In the hypoxia-stimulated condition, the MTT assay demonstrated significant cell-killing efficacy of PCF-a NEs on the 4T1 cell line, recording values of 94.4% and 94.2% under US and laser irradiation, respectively (808 nm). The alloy nanozyme, with good biocompatibility, high stability, no side effects on normal tissue, and excellent photothermal transformation efficiency (about 62%), can be a good candidate for tumor-specific therapeutic aims. *In vivo,* anticancer efficacy of different treatment groups, including PBS, (b) PCF-a NEs, (c) PCF-a NEs + US, and (d) PCF-a NEs + laser on 4T1 tumor-bearing mice, have confirmed remarkable tumor growth inhibition of PCF-a NEs with 5 min laser irradiation via photothermal and enhanced chemodynamic therapy. 

Qian *et al*. (91) developed an exceptional Fe-N-C nanoagent at the single-atom level. This was achieved by subjecting dimethylimidazole and iron (III) nitrate to a pyrolysis process in cu-nanoagent, which exhibited remarkable antitumor properties. Under 808 nm laser irradiation, the nanozyme increased the temperature from 28 °C to 56 °C for photothermal cancer therapy. It had stable photothermal capability, and the maximum produced temperature by the nanoagent was constant over time. The nanoagent exhibited a coordination framework akin to that of the peroxidase enzyme due to the presence of Fe atoms in its central coordinating region(92). What is intriguing is that it has the potential to function as an effective chemotherapeutic agent by catalyzing H_2_O_2_ into hydroxyl radicals without requiring NIR irradiation. The nanozyme, with the cooperation of laser irradiation, reached a higher than 90% decrease in the 4T1 cancer cell viability, which was more efficient than monotherapy by nanozyme function or laser exposure. The Fe-N-C single-atom nanoagent could cause cancer cell apoptosis by impairing mitochondrial OXPHOS, raising glycolysis, and generating reactive oxygen species.

**Figure 9:**
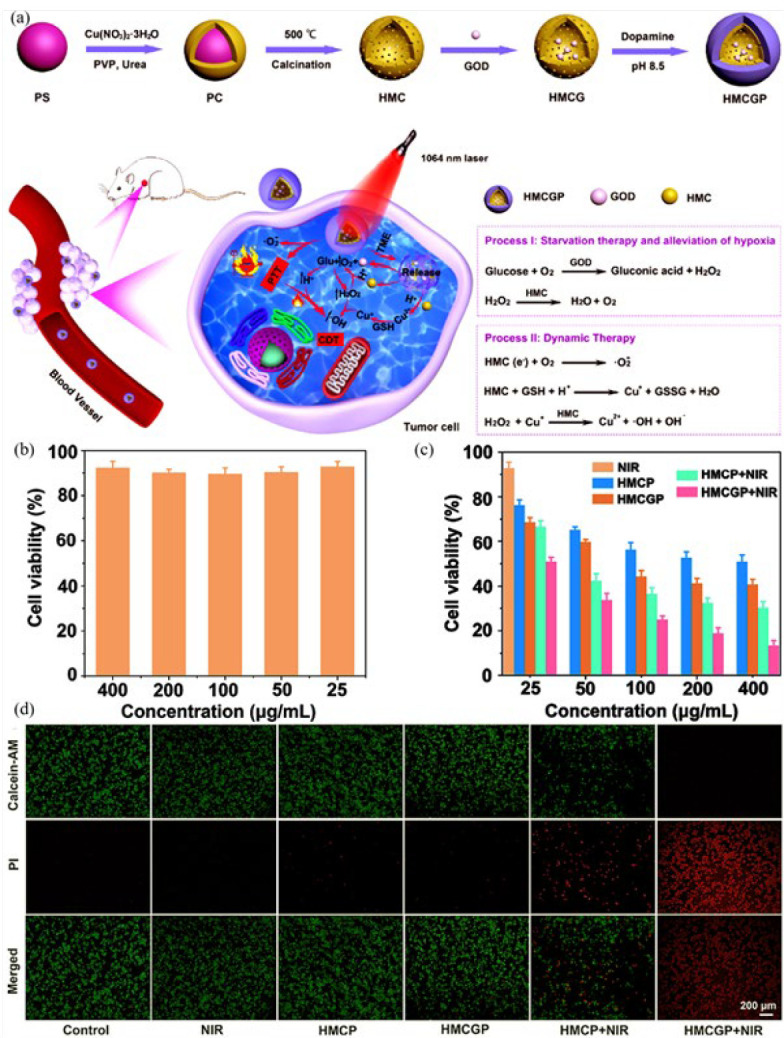
(a) Schematic indication of construction and function of hollow CuO nanosphere for cancer therapy. A hollow mesoporous nanosphere (HMC) was formed on a PS template. A substantial amount of glucose oxidase (GOD) was incorporated into the nanosphere, creating an HMCG nanozyme. Then, it was covered by polydopamine to have potent photothermal efficiency. After injecting into the tumor cells, GOD proceeded with the oxidation of glucose, which obtained H_2_O_2_ for further chemotherapy. Also, HMC catalyzed H_2_O_2_ to O_2_ molecules to relieve tumor hypoxia. The accumulated gluconic acid disrupted HMC to Cu^2+^ ions. A reaction of glutathione (GSH) with HMC produced Cu^+^ ions for further generation of hydroxyl radicals as toxic reactive segments for chemodynamic therapy. (b) Cell viability of L929 after treatment with HMCGP for 24 hr. (c) HeLa cell viability after different treatments with NIR, HMCP, HMCGP, HMCP + NIR, and HMCGP + NIR. (d) Live/dead analysis of Hela cells treated with NIR, HMCP, HMCGP, HMCP + NIR, and HMCGP + NIR. Reproduced by permission from American Chemical Society (93).

Wang *et al*. (93) presented a hollow mesoporous copper oxide (HMC) with photothermal-catalytic activity (Figure 9). Carboxylated polystyrene NPs were used as a template to synthesize hollow nanospheres (HMC). The hollow nanospheres were loaded with glucose oxidase (GOD) and decorated with polydopamine as a superior biocompatible matter with excellent photothermal efficiency under NIR radiation to form (HMCGP). With high hydrophilicity and effective breakdown in the tumor environment, polydopamine was considered a promising candidate for modifying the hybrid nanozyme. The hollow nanosphere structure afforded a great loading rate of about 47.1% for glucose oxidase to achieve effective starvation therapy. The enzyme glucose oxidase facilitated glucose oxidation within the HeLa cancer cells, resulting in a substantial generation of H_2_O_2_ for subsequent chemotherapy treatments. Moreover, the catalytic function of the CuO nanosphere generated numerous O_2_ molecules to alleviate tumor hypoxia. Under 1064 laser (NIR-II) irradiation, a photothermal exchange rate of 30.2% was efficient for photothermal therapy. *In vivo* accumulation of gluconic acid degraded hollow CuO nanosphere, resulting in the release of Cu^2+^ ions. A high intracellular concentration of glutathione reduced Cu^2+^ to Cu^+^ ones, which proceeded to a Fenton-like reaction by involving H_2_O_2_ and producing toxic hydroxyl radicals for chemodynamic therapy. The introduced hollow CuO nanozyme with superior *in vitro* tumor therapy and brilliant *in vivo* tumor prevention rate (greater than 92.1%) is a potent synergistic photothermal and chemodynamic cancer therapy agent. The Biocompatibility of HMCGP was confirmed through incubation with L929 fibroblast cells for 24 hr, and GSH and H_2_O_2_ expression was low in the normal cells (Figure 9b); the HMCGP plus NIR group exhibited a significant and impressive ability to combat cancer in HeLa cells compared to the other groups. This was evident from the remarkably low cell viability of only 11.6% at a concentration of 400 μg/ml, indicating a powerful combined therapeutic effect (Figure 9c). The therapeutic effect of HMCGP and NIR irradiation on the HeLa cells was also confirmed through Calcein-AM and PI (Propidium iodide) co-staining fluorescence images (Figure 9d). Besides, detecting very little Cu in the liver, heart, lung, spleen, and kidney showed its excellent biosafety.

## Photothermal-photodynamic assays

Photodynamic therapy, a conventional oxygen-reliant technique, operates by utilizing photosensitizers and generating reactive oxygen species via the conversion of NIR. This process induces apoptosis and necrosis, ultimately working towards antitumor objectives (94, 95). The synergic effect of the photothermal and photodynamic agents improves the cancer treatment potency by allowing deeper penetration and minimal invasiveness (96). 

Zhang *et al*. (97) synthesized carbon-gold nanohybrid for diagnosis and treatment based on photothermal and photodynamic methods. With the benefits of uniform size, good biocompatibility, high surface area, and heat generation under NIR light radiation, mesoporous carbon nanospheres were used to construct the nanozyme. The mesoporous nanozyme (OMCAPs) was obtained by doping mesoporous carbon nanospheres (MCNs) with small AuNPs and treatment with sulfuric and nitric acid. OMCAPs with abundant carboxyl groups on their surface were produced to increase hydrophilicity and stabilized by a mixture of folic acid and serum albumin (rBSA-FA). Folic acid was utilized to improve the targeted yield of the nanozyme in the gastric tumor cells. In cooperation with folic acid, serum albumin provided numerous anchoring sites for IR780 iodide fluorescent dye to obtain OMCAPs@rBSA-FA@IR780 nanoprobes that made it suitable for real-time, and NIR imaging as well as photothermal therapy under 808 nm laser irradiation. By increasing the temperature to 52.2 °C, the synthesized nanozyme with a strong photothermal effect could treat the cancer cells. Through entering the MGC-803 tumor cells (gastric carcinoma cell line), AuNPs of the nanozyme facilitated the catalytic oxidation of H_2_O_2_. Consequently, this process induced hydroxyl radical production, eradicating tumor cells. In addition, the nanohybrid exhibited a remarkable ability to generate ^1^O_2_ molecules by transferring energy from excited states to O_2_ molecules, thereby demonstrating its excellent photodynamic functionality. The nanozyme ablated tumor cells after 30 days and was ideally involved in cancer therapy. Furthermore, no apparent pathological harm or irregularity was detected in vital organs such as the heart, liver, spleen, lungs, and kidneys.

Xu *et al*. (98) presented a nanozyme using ruthenium (IV) oxide (RuO_2_) and bovine serum albumin (BSA) for photothermal and photodynamic therapy of hypoxic tumors. BSA was chosen based on superb colloidal stability, outstanding biocompatibility, and excellent loading capacity of photosensitizers and drugs. The RuO_2_@BSA nanosphere was synthesized using a repeatable and facile bio-mineralization strategy without toxic reagents. After that, a large amount of NIR photosensitizer (IR-808-Br_2_) was anchored on the BSA shell, and the robust RuO_2_@BSA@IR-808-Br_2 __(RBIR)_ nanozyme was produced for cancer phototherapy. After an injection into the 4T1 tumor-bearing mice, the nanozyme as a photothermal agent generated O_2_ molecules under 808 nm laser irradiation, and then, the anchored NIR photosensitizer converted them to cytotoxic ^1^O_2_ under laser irradiation. The inhibition tumor rate of RBIR reached above 90% by the nanozyme, making it promising for clinical applications, and showed the augmented synergistic effect of combination therapy. The MTS assays demonstrated the cytotoxicity of RBIR, revealing that the cell proliferation for each concentration group against 4T1 in the absence of light exceeded 85%. This signifies an exceptional level of biocompatibility for the developed nanozyme under dark conditions. Besides, no apparent tissue abnormalities or inflammatory lesions were found in the heart, liver, spleen, lung, and kidney, proving the negligible systemic toxicity of the nanozyme.

Researchers (99) successfully created a nanozyme by combining Pt-carbon materials. This innovative approach demonstrated excellent catalase-like activity while also harnessing photosensitizing properties. The researchers observed an enhanced synergistic effect in their experiments. At first, ZIF-8 (as a metal-organic framework) was covered by SiO_2_ to form ZIF-8@SiO_2_. Next, through etching and carbonization mechanism, carbon nanozyme was constructed from ZIF-8@SiO_2, _which NaBH4 further reduced in the presence of H_2_PtCl_4 _to form Pt-carbon nanozyme. The nanozyme was synthesized through a reduction method by immobilizing PtNPs on a carbon-coated metal-organic framework (MOF) skeleton. PtNPs improved the catalase-like function and photothermal performance of the nanozyme. In mouse colon tumor tissues, PtNPs catalyzed the over-existing H_2_O_2_ to O_2_ molecules for further photodynamic therapy by producing reactive oxygen species under 808 nm laser irradiation exposure. The Pt-carbon nanozyme efficiently inhibited tumor cell growth above 90%, significantly greater than the carbon nanozyme with 54%. The nanozyme successfully induced the inhibitory effect on the CT26 cell growth. Additionally, the Pt-carbon integrated nanozyme exhibited a photothermal conversion efficiency of 39%, while the carbon nanoagent demonstrated a slightly lower efficacy at 32%. The synthesized nanozyme exhibited a superior photothermal conversion capability, as suggested. The successful initiation of tumor cell apoptosis, tumor growth inhibition, and tumor necrosis induction were observed in various treatment groups by applying hematoxylin and eosin (H&E), TUNEL, and Ki67 staining techniques. Notably when comparing Pt-carbon nanozyme + NIR to other treatment groups. In relation to the absence of significant adverse reactions in healthy mice, the Pt-carbon nanozyme emerges as an innovative and appropriate tool for tumor treatment approaches.

Yang *et al*. (100) applied hollow nitrogen-doped carbon nanospheres (HNCSs) and iron phthalocyanine (FePc) for the synthesis of FePc/HNCSs nanozyme with both peroxidase-like and catalase-like activities. Firstly, SiO_2_@HNCSs were constructed through pyrolysis of The SiO_2_@polymer spheres. Then, the SiO_2_@HNCSs formed the HNCSs by etching SiO_2_ in the presence of NaOH at 70 °C. The final compound (FePc/HNCSs) was achieved from a mixture of HNCSs and FePc in a DMF solution. FePc component significantly improved the catalytic function of carbon nanospheres for efficient dual phototherapy. The peroxidase- and catalase-like activities of the nanozyme were mainly dependent on the FePc segment, while its photothermal and photodynamic functions originated from HNCSs. The peroxidase function of the FePc/HNCSs nanozyme resulted in numerous hydroxyl radicals, which directly destroyed tumor cells. Under light irradiation, the uptake of FePc/HNCSs into the 4T1 cell line reached 42.1%. Relying on its catalase function, the nanozyme generated O_2_ molecules from H_2_O_2_ for further photodynamic therapy of cancer cells. Under the 808 nm NIR irradiation, it achieved a great photothermal conversion efficiency of 38.7%. The dual phototherapy and catalytic therapy of FePc/HNCSs nanozyme led to an outstanding 4T1 tumor prevention rate of 96.3%. 

**Figure 10:**
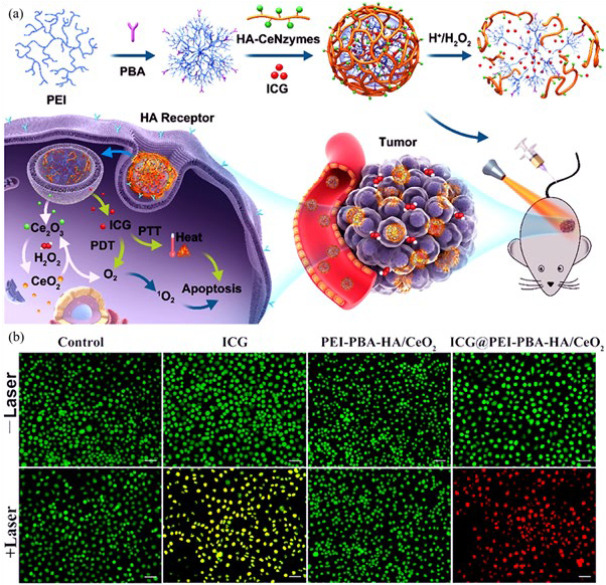
(a) Schematic demonstration of synthesis and therapeutic function of PEI-PBA-HA-CeO_2_ nanozyme. PEI-PBA attachments were used as the anchorage site for ICG photosensitizer molecules. HA provided sites for binding CeO_2_ nanozyme. The HA receptors on the cancer cells induced the targeted transfer of the ICG-loaded nanozyme to the cells. In the tumor cells, the PEI was depolymerized, and ICG molecules were released. Hence, the intracellular temperature increased (photothermal effect), and 1O_2_ components (photodynamic effect) were produced for cell apoptosis. (b) Calcein-AM/PI staining of MCF-7 cells after different treatments. Green spot stained with Calcein-AM and red spot stained with propidium iodide (PI) show viable and dead/apoptotic cells, respectively. Reproduced by permission from American Chemical Society (101).

A study (101) synthesized cerium oxide (CeO_2_) nanozyme-attached hyaluronic acid (HA) nanocage filled with indocyanine green (ICG) photosensitizer for tumor therapy. Figure 10 depicts the construction and function process of the nanozyme. ICG molecules were anchored by combining 4-carboxylphenylboronic acid pinacol ester (PBA) and polyethyleneimine (PEI), which were used to create scaffolds. Finally, the connection between PEI-PBA and HA-CeO_2_ nanozyme was established through a condensation reaction involving cis diol and boric acid groups. In the next step, ICG was added to the aqueous solution, and finally, ICG@PEI-PBA-HA/CeO_2_ was constructed. After receiving the tumor cells, the HA receptors on the cell surface caused the effective targeting of the ICG-loaded nanozyme. The pH-dependent cleavage of PBA released CeO_2_, which regulated the cancer’s hypoxic environment. With depolymerization of PEI, ICG molecules were released for laser-mediated photothermal, photodynamic therapy, and cell apoptosis in MCF-7 (breast cancer cell line) cancerous tissues under 808 nm laser irradiation exposure. After different treatments, it was shown that significant apoptosis and therapeutic efficacy were observed only in the ICG@PEI-PBA-HA/CeO_2_ + laser group (Figure 10b). The tumor tissues in the mice treated with nanozyme completely disappeared after 15 days, highlighting the good therapeutic effect of the nanozyme on tumors. Furthermore, a minor increase in liver content was detected; however, no noteworthy harm was witnessed. With excellent stability, simple production, and flexible composition, the PEI-PBA-HA-CeO_2_ nanozyme is promising for cancer therapeutic assays. 

Xie *et al*. (102) fabricated an efficient therapeutic nanozyme by using Pt and bismuth (Bi) elements, β-cyclodextrin (β-CD), and Ce6 molecules in cancer therapy. β-CD molecules with numerous hydrophobic cavities and hydroxyl groups were immobilized on the surface of a hexagonal PtBi plate through host-guest interactions, which provided great attachment sites for the Ce6 photosensitizer molecules and photodynamic process. The nanozyme’s size (101.3 nm) remained unchanged following a week of incubation, indicating its remarkable stability. After an injection to U14 tumor-bearing Balb/c mice, the photothermal conversion efficiency was obtained at 35.9% and 39.9% under 808 nm and 1064 nm laser irradiation, respectively. This issue highlighted the excellent photothermal response of the PtBi-β-CD-Ce6 nanozyme. The approximately constant temperature during four laser irradiations confirmed the excellent photothermal stability of the nanozyme. On the PtBi surface, H_2_O_2_ adhered and completely covered it due to more advantageous thermodynamic adsorption energy than that required for H_2_O. Consequently, the nanozyme PtBi-β-CD-Ce6 exhibited catalase activity, producing O_2_ molecules that facilitated the generation of ^1^O_2_ as an apoptosis agent. Under laser irradiation, Ce6 photosensitizer induced photodynamic therapy of the nanozyme by producing ^1^O_2_ molecules. The nanozyme could effectively prevent tumor growth, while normal morphology of many organs without extensive damage was observed after exposure to the nanozyme, indicating its excellent biosafety profile. The nanozyme was also efficient for photothermal and photoacoustic imaging. 

Li *et al*. (103) introduced a bimetallic nanozyme by using AuNPs and RuNPs anchored on a dendritic mesoporous skeleton of silica (DMSN-Au@Ru NPs) using the synergistic effect of self-enhanced PDT and PTT. AuNPs with excellent oxidase-mimicking properties were used to catalyze the oxidation process of glucose for H_2_O_2 _production. RuNPs were applied to generate ^1^O_2_ reactive species by decomposing H_2_O_2_. Therefore, nanozymes can mitigate tumor hypoxia and attain a powerful photodynamic impact. It was shown that the constructed nanozyme did not have a noticeable effect on cell viabilities of HeLa, 4T1, and 3T3, indicating good biocompatibility of the nanozyme. Interestingly, after exposure to HeLa cells for 24 hr and 808 nm laser irradiation, the nanozyme could kill more than 90% of cells. Inspired by an *in vitro* assay, a U14 tumor-bearing mice model was performed to investigate the therapeutic effect of the nanozyme. The DMSN-Au@Ru NPs represented the impressive photothermal effects upon 808 nm laser irradiation (55 °C within 10 min) relying on the intense absorption of RuNPs in the NIR region. Besides, it caused no noticeable damage to the principal organs of the treated mice (heart, liver, spleen, lung, and kidney), indicating its excellent biocompatibility and non-toxicity.

Kang *et al*. (104) synthesized MoS_2_-Co_3_S_4_@PEG nanoflowers (MSCs@PEG) to achieve NIR-II-triggered photothermal and photodynamic cancer therapy. MoS_2_ nanoflowers were synthesized by a hydrothermal process, and then, Co_3_S_4_ nanodots were loaded on their surface. The nanozyme attained a photothermal conversion rate of 39.8% in the cancer tissues upon 1064 nm NIR-II irradiation. Also, hyperpyrexia could provide extra energy for the co-excitation of Co_3_S_4_ (1.40 eV) and MoS_2_ (1.14 eV) under a low-energy NIR-II laser (1.16 eV). The Z-scheme mechanism of MSCs nanoflowers represented the appropriate redox capability for oxidizing water and simultaneously forming O_2 _and reactive oxygen species. The MSCs@PEG nanozyme converted H_2_O_2_ to hydroxyl radical and O_2_ molecules for hypoxia relief and chemotherapy, relying on its peroxidase-like and catalase-like function. After different treatments, only the MSC-2@PEG+NIR-II group demonstrated a noticeable decrease in tumor size. H&E staining of tumor tissue also confirmed considerable necrosis and atrophy due to combination therapy. Since immunogenic cell death (ICD) could assist the maturation of dendritic cells (DCs) for arousing antitumor immune response, the inguinal lymph of 3 days-treated mice was gathered, and lymphoid CD80 and CD86 levels were analyzed. The DC-maturated percent for the nanozyme with NIR-II component was 19.8%, more than that for the control sample (9.8%). The TNF-α, IL-2, and IFN-γ cytokine levels in the MSCs@PEG exposed cells indicated a significant increase compared to the control group. These cytokines serve as indicators for cellular immunity, humoral immunity, and secretions characteristic of Th1 cells. Specifically, their concentrations were 1.6 times higher for TNF-α, 1.2 times higher for IL-2, and 3.8 times higher for IFN-γ than those observed in the control group. It proved the nanozyme’s potential to activate the immune system for anticancer proceedings.

## Conclusion and future perspective

This research presents the latest advancements in tumor therapy, specifically focusing on photothermal and photothermal-combined techniques. A diverse range of nanozyme structures further enhances these innovative approaches. Photothermal therapy generates drastic thermal damage in targeted cancerous tissues as a safe therapeutic approach by controlling the irradiation parameters. 

Promising photothermal nanomachines encompass metallic nanoparticles, bimetallic nanosubstances, single-atom nanozymes, and metal oxide nanoagents. 

These entities demonstrate high selectivity along with low toxicity and noninvasive side effects. Besides, photothermal nanozymes can be improved to induce effective multi-therapeutic functions. This technique is minimally invasive, diminishing the risks typically associated with conventional surgical methods. Additionally, PTT is beneficial, as it operates separately from systemic pharmacological agents, thereby avoiding problems related to drug resistance. However, its effectiveness is mainly restricted by the limited light penetration depth, making it less appropriate for treating deep tumors. Additionally, the activation of heat shock proteins in tumor cells can provide a protective mechanism against thermal injury, causing a possible obstacle to the effectiveness of treatment. The compounds mentioned in this review include innovative nanoenzymes with photothermal properties, which perform their effect through the synergy of their enzymatic abilities and photothermal capabilities, which pose their effects through the combined action of their enzymatic functions and photothermal properties. These compounds effectively lead to the removal of over 80% of cancer cells, reaching as high as 97% (67). They exhibit a photothermal conversion range of about 35–45% and have high selective permeability to penetrate cancerous tissues. The primary excretion of these substances is from the liver, and their accumulation in the liver is more than in other body organs. All of the compounds have shown little toxicity among healthy cells, and they have not had debatable adverse effects. Their activation and performance are entirely dependent on laser radiation due to their design method and inherent characteristics, which is the reason for their high specificity. These substances generally lead to an increase in the temperature of the tumor area to about 50 to 55 degrees. In some cases, the temperature has reached 60 degrees (61, 64).

As one of the noninvasive cancer treatment assays, sonodynamic therapy combines low-intensity ultrasound and sonosensitizer to combat cancer by releasing energy and producing reactive oxygen species. The synergistic effect of photothermal and sonodynamic therapy results in complete cancer eradication with no evidence of recurrence. Until now, various substances have been utilized as sonosensitizers, including compounds derived from porphyrin, xanthene, non-steroidal medications, and other similar sources. Erythrosin B and Rose Bengal, derived from xanthene compounds, exhibit remarkable effectiveness as sonosensitizers. However, their drawbacks encompass rapid sequestration within the liver and insufficient accumulation in cancerous tissues. HMME, PpIX, and Ce6 molecules as porphyrin-based sonosensitizers have been applied in cancer therapy. The occurrence of skin phototoxicity caused by HMME and PpIX hinders their suitability for clinical use (105). Ce6, with selective accumulation in cancer tissues and rapid clearance from normal cells, has been extensively utilized as a sonosensitizer (106). Recently, Ce6 molecules in a hydrogel scaffold with good encapsulation ability were applied for photothermal/sonodynamic therapy, indicating superior potential for clinical purposes (77). There are different non-steroidal drugs with anticancer effects under ultrasound irradiation, such as ciprofloxacin, lomefloxacin, sparfloxacin, and gatifloxacin. In terms of future prospects, a combination of diverse sonosensitizers and hydrogel carriers holds immense potential in nanozyme therapy. Moreover, nanomaterials with good sonosensitivity properties are promising in photothermal/sonodynamic efficient therapy. There are various nanomaterials as potent nanosonosensitizer for photothermal/sonodynamic therapy, including polymeric, inorganic, noble metal, liposomal, and carbon-based ones (107, 108).

Recently, a novel photothermal/sonodynamic therapy approach has emerged using hollow Pt-CuS Janus nanozyme. This innovative technique holds great promise for achieving highly efficient sonodynamic cancer treatment. Introducing diverse composites derived from various nanomaterials adds further novelty to this therapeutic strategy. PTT hire photothermal agents to convert light energy into heat, inducing localized hyperthermia and promoting apoptosis in tumor cells. On the other hand, SDT utilizes ultrasound to activate sonosensitizers, producing reactive oxygen species that pose oxidative damage to cancerous cells. The hyperthermic environment created by PTT increases cellular membrane permeability and facilitates deeper penetration of ultrasound waves, thereby amplifying the activation of sonosensitizers. This synergistic interaction can improve tumor ablation and a more robust immune response. Combining these two methods, increasing the temperature and creating the sonodynamic cavitation, increases the enzyme activity and leads to the efficient removal and destruction of cancer cells. The liver and spleen accumulated more particles, while in hydrogel-based nanozyme, more particles accumulated in the spleen. The effect of hydrogel-based nanozyme is time-dependent, whereas non-hydrogel compounds show a dose-dependent effect. Hydrogel-based nanozymes result in lower tumor temperature increases and more uniform particle dispersion in the body.

As another promising noninvasive cancer treatment technique, photodynamic therapy can use light radiation with a light-activated chemical (photosensitizer) to generate reactive oxygen species in cancerous cells. Mesoporous carbon-gold hybrid (97), metal oxides-included protein nanoagents (98), hollow nitrogen-doped carbon nanocages (100), gold/ruthenium-mesoporous silica (103), cerium oxide-loaded hyaluronic acid [86], metal-involved β-cyclodextrin (102), and MoS2 nanoflowers (104) are known as efficient photosensitizers. Combining PTT with photodynamic therapy (PDT) could take advantage of the inherent benefits of both techniques to enhance treatment. PTT-induced localized hyperthermia enhances the uptake and activation of photosensitizers utilized in PDT. Moreover, the elevation in temperature enhances tumor perfusion and oxygenation, which is critical for the efficacy of PDT, given its dependence on oxygen for ROS generation. This synergistic strategy can lead to enhanced effectiveness in tumor cell cytotoxicity and decreased tumor recurrence rates. Moreover, this combined approach can stimulate the immune system, enhancing systemic antitumor responses. As a future aim, upconversion nanoparticles with the conversion capability of low-energy photons to high-energy emission can be applied for efficient photodynamic/photothermal therapy (111). Also, integrating diverse nanomaterials into polymeric compounds can yield enhanced robust photostability, great light-conversion ability, and smart modifiable drug carriers (112). Ultimately, this innovation offers a groundbreaking solution to combat cancer using photodynamic/photothermal techniques effectively. 

 Chemodynamic therapy is an emerging cancer treatment that converts endogenous H_2_O_2_ molecules to harmful hydroxyl radicals. The effectiveness of chemodynamic therapy is hindered by insufficient hydroxyl radicals and a sluggish production rate. Combining PTT with chemodynamic therapy (CDT) also exploits the distinct mechanisms of both treatments. PTT-induced hyperthermia could boost the catalytic activity of chemodynamic agents, thereby enhancing ROS production in CDT. The thermal effects may alter the tumor microenvironment, creating conditions more conducive to ROS generation via CDT. Such a combination can yield superior tumor destruction and address the limitations associated with each therapy when administered in isolation. This combined approach also allows for a reduction in the dosage of chemodynamic agents, which is likely to result in lower toxicity and fewer adverse effects. Until now, various inorganic nanomaterials containing transition metals and metal oxides have proven advantageous for chemodynamic cancer treatment. These materials strengthen Fenton and Fenton-like reactions that aid in eradicating cancer cells. Examples include iron, silver, gold, ruthenium, copper, palladium, cerium oxide, and vanadium oxide, among others. Interestingly, Cu^+^ ions accelerated the generation of hydroxyl radicals about 160 times more than Fe^2+^ (113)o D. Furthermore, through the utilization of diverse nanocomposites, they will contribute to the advancement of efficient strategies for tumor treatment. The yolk-shell nanostructures (89), ZIF-8 (114), MIL-100 (83), MOF skeletons, and carbon dots (84) have been applied as efficient frameworks for the construction of potent therapeutic nanozymes. However, other skeletons can be suitable for chemodynamic/photothermal therapy, such as UiO-66 and HKUST-1 MOFs, covalent organic frameworks (COFs), MXene nanocomposites, carbon-based nanosheets, metal core-shell nanoscaffolds, and so on. 

 To summarize, emphasizing highly efficient nanozymes with innovative designs offers an impactful approach to enhancing clinical cancer therapy.

SAzyme was synthesized based on the striping of single atoms from metal nanostructures. The obtained PdNPs were encapsulated into the ZIF-8 skeleton, producing Pd@ZIF-8 nanocomposite. Pd SAzyme was formed by pyrolysis of Pd@ZIF-8 nanocomposite at 900 °C. After its PEGylation, the SAzyme was completed to inject into BALB/c mice with 4T1 cancerous cells. Under 1064 nm laser irradiation, the nanozyme induced efficient photothermal conversion. Relying on its glutathione oxidase activity, a significant number of glutathione disulfide (GSSG) was produced from glutathione (GSH), which converted to lipid peroxides (LOP) for ferroptosis. Peroxidase-mimicking of Pd SAzyme also achieved many hydroxyl radicals for further ferroptosis. Reproduced with permission from John Wiley and Sons (58).

MIL-100 was used as a carrier for ABTS. In the presence of H_2_O_2_, the nanozyme activity of the nanoreactor was triggered and amplified in the acidic conditions of tumor cells. The peroxidase-like activity of MIL-100 converted ABTS to the oxidized form in the presence of H2O2, which is efficient for photoacoustic imaging. The nanoreactor generated hydroxyl radicals via the Fenton reaction, interacting with ferrous ions from MIL-100, facilitating cancer cells' photothermal therapy. The generated hydroxyl radicals were efficient for chemodynamic therapy. Reproduced by permission from Jon Wiley and Sons (83).

## Declaration

We have not used any AI tools or technologies to prepare this manuscript.
